# Exploring the destructive synergy between IL-33 and Suilysin hemolysis on blood-brain barrier stability

**DOI:** 10.1128/spectrum.00612-24

**Published:** 2024-07-09

**Authors:** Yang Fu, Jing Jie, Liang Lei, Mengmeng Liu, Junjie Wang, Liancheng Lei, Hongtao Liu

**Affiliations:** 1 State Key Laboratory for Diagnosis and Treatment of Severe Zoonotic Infectious Diseases, Key Laboratory for Zoonosis Research of the Ministry of Education, College of Veterinary Medicine; Department of Respiratory Medicine, Center for Pathogen Biology and Infectious Diseases, Key Laboratory of Organ Regeneration and Transplantation of the Ministry of Education, The First Hospital of Jilin University, Jilin University, Changchun, China; 2 Key Laboratory of the Provincial Education, Department of Heilongjiang for Common Animal Disease Prevention and Treatment, College of Veterinary Medicine, Northeast Agricultural University, Harbin, China; 3 Department of Orthopaedics, Heilongjiang Provincial Hospital, Harbin, Heilongjiang, China; Uniwersytet Medyczny w Bialymstoku, Bialystok, Poland

**Keywords:** *Streptococcus suis *type 2, Suilysin, blood-brain barrier, hemolytic products, IL-33

## Abstract

*Streptococcus suis* type 2 (SS2) is a zoonotic pathogen capable of eliciting meningitis, presenting significant challenges to both the swine industry and public health. Suilysin (Sly), one of SS2 most potent virulence determinants, releases a surfeit of inflammatory agents following red blood cell lysis. Notably, while current research on Sly role in SS2-induced meningitis predominantly centers on its interaction with the blood-brain barrier (BBB), the repercussions of Sly hemolytic products on BBB function have largely been sidestepped. In this vein, our study delves into the ramifications of Sly-induced hemolysis on BBB integrity. We discern that Sly hemolytic derivatives exacerbate the permeability of Sly-induced *in vitro* BBB models. Within these Sly hemolytic products, Interleukin-33 (IL-33) disrupts the expression and distribution of Claudin-5 in brain microvascular endothelial cells, facilitating the release of Interleukin-6 (IL-6) and Interleukin-8 (IL-8), thereby amplifying BBB permeability. Preliminary mechanistic insights suggest that IL-33-driven expression of IL-6 and IL-8 is orchestrated by the p38-mitogen-activated protein kinase signaling, whereas matrix metalloproteinase 9 mediates IL-33-induced suppression of Claudin-5. To validate these *in vitro* findings, an SS2-infected mouse model was established, and upon intravenous administration of growth stimulation expressed gene 2 (ST2) antibodies, *in vivo* results further underscored the pivotal role of the IL-33/ST2 axis during SS2 cerebral invasion. In summation, this study pioneerly illuminates the involvement of Sly hemolytic products in SS2-mediated BBB compromise and spotlights the instrumental role and primary mechanism of IL-33 therein. These insights enrich our comprehension of SS2 meningitis pathogenesis, laying pivotal groundwork for therapeutic advancements against SS2-induced meningitis.

**IMPORTANCE:**

The treatment of meningitis caused by *Streptococcus suis* type 2 (SS2) has always been a clinical challenge. Elucidating the molecular mechanisms by which SS2 breaches the blood-brain barrier (BBB) is crucial for the development of meningitis therapeutics. Suilysin (Sly) is one of the most important virulence factors of SS2, which can quickly lyse red blood cells and release large amounts of damage-associated molecular patterns, such as hemoglobin, IL-33, cyclophilin A, and so on. However, the impact of these hemolytic products on the function of BBB is unknown and ignored. This study is the first to investigate the effect of Sly hemolytic products on BBB function. The data are crucial for the study of the pathogenesis of SS2 meningitis and can provide an important reference for the development of meningitis therapeutics.

## INTRODUCTION

*Streptococcus suis* type 2 (SS2), a zoonotic pathogen, conspicuously stands out for its capability to incite meningitis, selectively targeting neonatal and weaned piglets and manifesting through neurological perturbations and motor impairments (1). SS2 not only confines its deleterious impacts to swine but also emerges as a formidable pathogen to humans, marking its stamp as one of the pivotal bacterial agents contributing to human bacterial meningitis. Cases of SS2 infections have breached the species barrier to influence humans globally. Noteworthy are the outbreaks that unfurled in Jiangsu and Sichuan provinces of China in 1998 and 2005, respectively. Which culminated in the infection of 240 individuals, tragically spiraling into 52 mortalities (2, 3). Consequently, SS2 meningitis not only inflicts significant economic depreciations onto the swine industry but also looms as a grave menace to public health safety. SS2 orchestrates its nefarious influence by infiltrating brain microvascular endothelial cells (BMEC) and subsequently trespassing the blood-brain barrier (BBB) to instigate cerebral infections within the central nervous system (CNS) (4, 5). However, a formidable challenge looms in the clinical sphere, as the antimicrobial agents conventionally deployed against cerebral bacterial invasions stymied by the BBB, rendering them impotent in achieving efficacious drug concentrations at target locales, and thereby mitigating the therapeutic outcomes against bacterial meningitis (6). The delineation of the pathogenic machinations wielded by SS2 stands imperative in sculpting effective preventative and therapeutic strategies against SS2-induced meningitis.

Suilysin (Sly), a pivotal virulence factor of SS2, intricately orchestrates its role during SS2 infection and host cell invasion, embodying a critical element in the disease pathogenesis. Sly, adept in resisting the phagocytic and bactericidal activities of neutrophils, coerces the cytoskeletal rearrangement in epithelial cells, enhancing SS2 adhesion and invasion capabilities and facilitating the pathogen dissemination at the infection locus (7). Beyond this, Sly induces inflammatory damage within the host and plays an influential role in SS2-induced meningitis. Sly modulates the Toll-like receptor 4 (TLR4), mitogen-activated protein kinase (MAPK), and phosphoinositide 3 kinase (PI3K) signaling pathways, leading to an exacerbated release of tumor necrosis factor-α (TNF-α) and heparin-binding protein, subsequently precipitating the inflammatory damage induced by SS2 infection (8). Furthermore, Sly can trigger TLR4-dependent inflammatory responses and platelet aggregation, culminating in the onset of SS2 toxic shock syndrome (9). Investigations spearheaded by our research group elucidate that post-infection with meningitis-inducing streptococcal strains, the secretion levels of Interleukin-6 (IL-6) from host lymphocytes are significantly elevated compared to those from non-meningitis-inducing streptococcal infection, with Sly emerging as one of the principal virulence factors precipitating the heightened expression of IL-6 during SS2 meningitis (10).

Research confirms that hemolytic products play a crucial role in the onset of numerous inflammatory diseases. Following hemolysis induced by Sly, red blood cells rupture, releasing abundant hemoglobin and damage-associated molecular patterns (DAMPs) into the bloodstream (11). Recognized DAMPs encompass Heme, HSP70, ATP, mitochondrial DNA (mtDNA), Interleukin-33 (IL-33), and Cyclophilin A (CypA), all of which are capable of inducing potent inflammatory responses within the host organism. Hemoglobin, in a cooperative interaction with bacterial cell wall components, stimulates macrophages, engendering the release of inflammatory cytokines such as Interleukin-1β (IL-1β), TNF-α, IL-6, and Interleukin-8 (IL-8). Concurrently, Heme is known to activate the nuclear factor kappa-B (NF-κB) signaling pathway, inducing vascular endothelial cells to express tissue factors and subsequently precipitating vascular inflammation, oxidative stress, and cellular apoptosis (12–14). IL-33, a nuclear member of the Interleukin-1 (IL-1) cytokine family, promotes the secretion of type II cytokines from innate immune cells via the growth stimulation expressed gene 2 (ST2) receptor, thereby furthering the activation of regulatory T cells among others (15). CypA, binding to the CD147 molecule on the cell surface, enhances the release of chemokines from inflammatory cells, inducing conditions such as rheumatoid arthritis, liver injury, and severe sepsis (16–18). mtDNA, activating the Toll-like receptor 9 (TLR9) pathway and fostering the formation of NLR family pyrin domain containing 3 (NLRP3) inflammasomes, participates in the occurrence of sterile inflammation (19, 20).

Penetration of the host BBB following bacteria infection represents a crucial juncture in the pathogenesis of subsequent meningitis (21). While existing research exploring the mechanisms of the involvement of Sly in SS2-induced meningitis primarily focuses on the interactions between Sly itself and the BBB, the influence of hemolytic products, released through Sly-mediated red cell lysis, on BBB functionality remains shrouded in scientific uncertainty. To address this, our study is centered Sly, hemolytic products, and the BBB. To elucidate the effects of Sly and hemolytic products on local BBB inflammation and barrier function, as well as their underlying molecular mechanisms, aiming to establish a theoretical foundation for the prevention and treatment of SS2-induced meningitis.

## MATERIALS AND METHODS

### Establishing a blood-brain barrier model *in vitro*

Adapting a method for constructing a BBB model from previously published procedures (22), with slight modifications by our team, the methodology employed is as follows: Place a Transwell in a 12-well cell culture plate and seed 20,000 hCMEC/D3 cells on the inner bottom surface of the Transwell. Concurrently, a DMEM control group is established. TEER value was monitored daily, and upon stabilization, it signifies successful model construction. Within this model, the upper chamber mimics the blood side of the BBB, while the lower chamber represents the brain side.

### Assessing barrier functionality in the *in vitro* BBB model

To investigate the impact of Sly and hemolytic products on BBB function in the *in vitro* BBB model, we utilized a prokaryotic expression system (BL21-pET28a::Sly) to express and purify Sly protein. Subsequent to this, the Toxin Eraser Endotoxin Removal Kit (Genscript, L00338) was utilized to eradicate endotoxins present within the Sly protein. Experimental groups were as follows: Sly treated group (with each well receiving 0.5 µM of Sly protein), RBC lysis treated group (10,000 RBCs lysed by sonication), Sly and RBC treated group (Sly/RBC), and an untreated control group. TEER values for each well in the respective groups were monitored every hour for a continuous 12 h duration, with the resultant TEER value fluctuations plotted as a curve.

### HRP permeability experiment

The BBB barrier functionality was assessed via the HRP permeability experiment (23). hCMEC/D3 cells were exposed to various treatments (Sly, RBC lysis, Sly/RBC, and untreated) for 12 h, followed by HRP (1 mg/mL) application to the upper chamber and 4 h incubation. Subsequent HRP quantification was conducted on the collected lower chamber medium.

Additionally, the impact of SS2 WT and SS2 Δsly on BBB barrier functionality, with RBC presence, analyzed through a similar methodology, utilizing groups: SS2 WT, SS2 Δsly, SS2 WT/RBC, SS2 Δsly/RBC, and untreated. Treatments for 6 h, HRP (1 mg/mL) was introduced, incubated for 4 h, and the lower chamber medium was analyzed for HRP content.

### qRT-PCR analysis

After treating hCMEC/D3 cells with various agents (Sly, RBC lysis, Sly/RBC, untreated), respectively, and at time intervals of 0, 2, 4, 6 h, culture media were discarded, cells were harvested, and total RNA was subsequently extracted. Quantitative RT-PCR to evaluate the transcription levels of cytokines, utilizing primers detailed in Table S1.

### ELISA analysis

Following a 6 h treatment of hCMEC/D3 cells under various conditions (Sly treated, RBC lysis, Sly/RBC, and untreated groups), culture media discarded and cells were collected. Cell lysis using NP-40, followed by centrifugation to gather the supernatant. Cytokine expression levels were then quantified using IL-6 (Biolegend, 430504), IL-8 (Biolegend, 431504) and MMP-9 (Beyotime, PM738) ELISA assay kits.

Employing signal pathway inhibitors, namely, the p38 inhibitor Adezmapimod (SB203580) (Selleck, S1076) and the MMP-9 inhibitor Marimastat (BB-2516) (Selleck, S7156), we conducted an ELISA to analyze the expression of IL-6 and IL-8 in the presence of IL-33.

### Western blot analysis

To assess the impact of IL-33 on the expression of Claudin-5, MAPK, and MMP-9, hCMEC/D3 cells were treated with various concentrations of IL-33 (Abcam, ab187455) (0.1, 0.5, 1, 10, 100 ng/mL) for 6 h. Subsequent to treatment, culture media discarded and cells harvested, lysed with NP-40, and centrifuged to retrieve the supernatant. Claudin-5 expression analyzed by Western blotting, employing a primary antibody (Mouse anti-Claudin-5) at a dilution of 1:3,000 and a secondary antibody of Alexa Fluor 680-labeled goat anti-mouse IgG (Abcam, ab175781). Analogous methodology was employed to assess the expression of p38 MAPK, phosphorylated p38 MAPK (p-p38 MAPK), and MMP-9 via Western blot analysis. Primary antibodies used included rabbit anti-Claudin-5 (Thermo Fisher, 35-2500), rabbit anti-p38 MAPK (Cell Signaling Technology, 8690), and rabbit anti-p-p38 MAPK (Cell Signaling Technology, 4511), each at a dilution of 1:3,000. The secondary antibody was Alexa Fluor 790-labeled goat anti-rabbit IgG (Abcam, ab175773).

### Immunofluorescence analysis

To elucidate the influence of IL-33 on Claudin-5 expression and localization, hCMEC/D3 cells were treated with 10 ng/mL of IL-33 for a duration of 6 h. Post-treatment, the cells were fixed using 4% paraformaldehyde and subsequently subjected to immunofluorescence staining. The primary antibody employed was Mouse anti-Claudin-5 (Thermofisher, 35-2500), used at a dilution of 1:200, while the secondary antibody was Alexa Fluor 488-conjugated Goat anti-mouse IgG (Abcam, ab150113). Nuclei stained utilizing Hoechst at a 1:10,000 dilution. Observations and image capture were executed utilizing an Olympus IX83 fluorescence microscope.

#### SS2 strain inoculation and mortality observation

C57BL/6 mice were intravenously injected with 1 × 10^7^ CFUs of log-phase SS2 strain (SC19). Prior to SS2 inoculation, mice received an intravenous injection of anti-ST2 (Astegolimab, Selleck, A2443) (1 µg in 100 µL saline) 30 min before the challenge. Mice were continuously monitored for behavioral changes over 96 h, and mortality was recorded.

#### BBB permeability and bacterial load assessment

At 6 h post-infection with the SS2 strain, the permeability of the mice BBB was examined using the Evans Blue (EB) permeability assay. Bacterial loads in both the blood and brain tissues of the mice were determined through plate counts. Blood samples were anticoagulated with sodium heparin and subsequently serially diluted for plate counting. Meanwhile, 1 g of brain tissue was homogenized in 500 µL of sterile PBS. The homogenate was then serially diluted and plated for bacterial counts.

#### IL-33 injection and BBB permeability examination

C57BL/6 mice were intravenously injected with IL-33 at dosages of either 10 ng or 100 ng in 100 µL saline per mouse. BBB permeability was assessed using the EB permeability assay 6 h post-injection.

### Evans Blue permeability assay

EB dye, under normal circumstances, cannot penetrate the BBB to enter the brain. However, in instances where the BBB compromised due to various pathological factors, EB can permeate into the brain, serving as a metric for assessing BBB integrity. In this study, the EB permeability assay was employed to evaluate the extent of BBB disruption induced by Sly hemolytic products and SS2 (24). Post-extraction of the brain, its wet weight was measured using an electronic balance and subsequently placed in a centrifuge tube containing 2 mL of formamide. The brain was then extracted in the dark at 37°C for 48 h to isolate EB. Following thorough mixing and settling, the supernatant was collected and absorbance measured at 620 nm. The content of EB in the brain was calculated utilizing a standard curve, with results expressed as the content of EB per gram of brain tissue.

### Statistical analysis

All data were subjected to analysis utilizing one-way ANOVA through GraphPad Prism 8.0 (Graph Pad Software, La Jolla, CA), with the sole exception being the data derived from the mouse survival assay. The experimental data pertaining to mouse survival were evaluated by using the log-rank test. *P* values are denoted in the figures as follows: **P < 0.01, *P < 0.05.

## RESULTS

### Sly hemolytic products augment Sly-induced disruption in an *in vitro* BBB model

**Fig 1:**
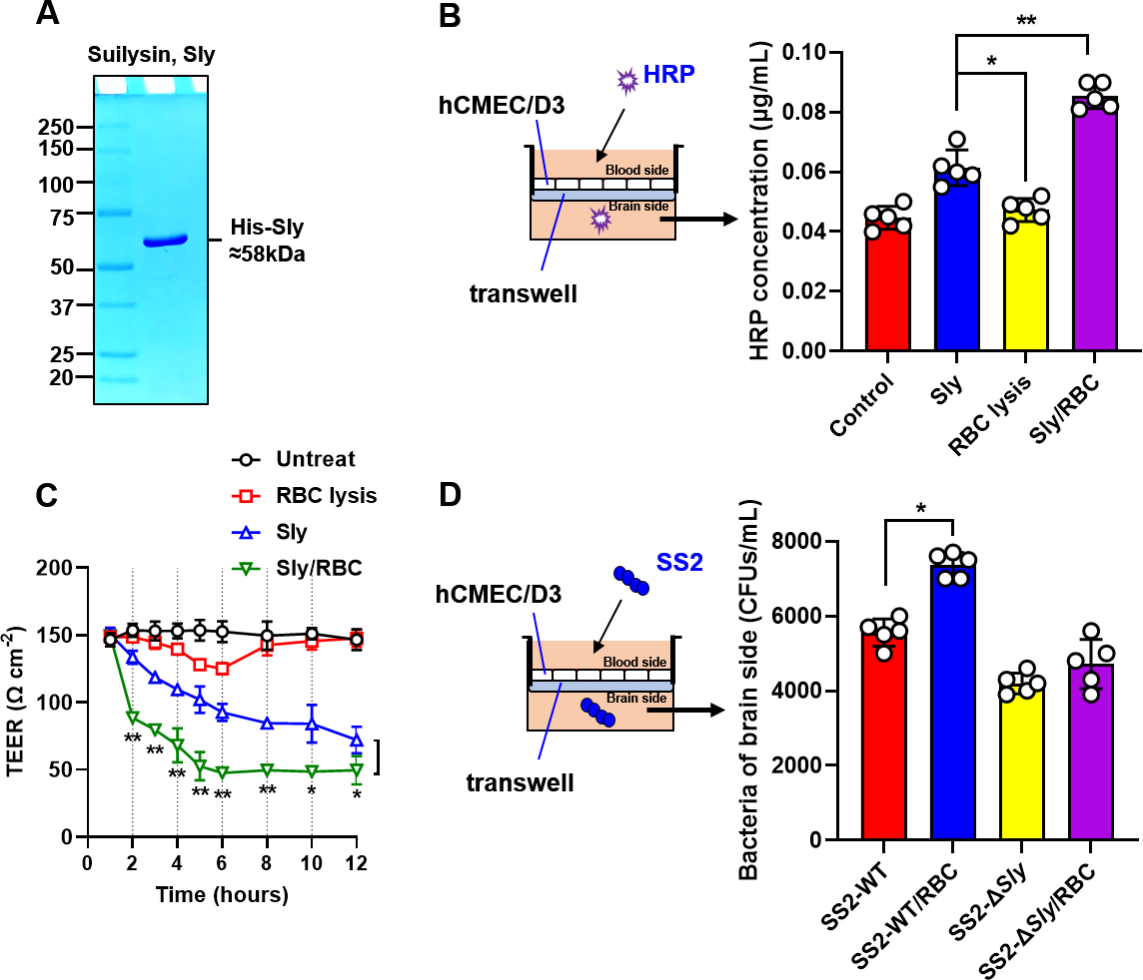
Sly hemolysin products amplify Sly-induced BBB model permeability. (**A**) SS2 Suilysin expression. Utilizing BL21-pET28a::Sly, Sly protein was expressed, purified using NI-NTA affinity chromatography, and endotoxins were eradicated. (**B**) HRP permeability assay investigating the impact of Sly hemolysin products on the BBB model. Post HRP addition to the upper chamber of the BBB model, the HRP concentration in the lower chamber was gauged following a 4-h interval. (**C**) TEER measurement to evaluate the impact of Sly hemolysin products on the BBB model (*n* = 3). (**D**) Assessing RBC impact on SS2-induced BBB model disruption using HRP permeability assay. RBC and SS2 strains, once mixed, added to the upper chamber of BBB model. The data are presented as the mean ± standard deviation (SD) and were analyzed by one-way ANOVA. **P < 0.01, *P < 0.05.

The ramifications of Sly and the ensuing hemolytic products, emerging from Sly-induced RBC lysis, on the functionality of the BBB remain undocumented. In this investigation, Sly protein was initially expressed and purified through a prokaryotic expression system (BL21-pET28a::Sly) (Fig. 1A). Thereafter, it co-incubated with RBCs in the BBB model, concurrently establishing groups administered with ultrasonically lysed RBC products, Sly alone, and an untreated control. The influence on the BBB model barrier functionality across all groups appraised employing an HRP permeability assay. Remarkably, relative to the untreated group, the BBB model subjected to Sly alone demonstrated heightened permeability to HRP at 12 h (Fig. 1B). In contrast to the Sly-only group, the group administered with both Sly and RBCs exhibited a significantly augmented permeability to HRP within the BBB model (Fig. 1B).

TEER value, reflective of barrier integrity, disclosed that, in comparison to the untreated group, the Sly treated group witnessed a noteworthy reduction in TEER commencing from 2 h, whereas the RBC lysis group displayed pronounced reductions at both 4 and 6 h, albeit TEER gradually reverted to baseline levels by 8 h (Fig. 1C). Intriguingly, the Sly and RBC treated group experienced a considerable decrement in TEER, initiating from 2 h, which was substantially lower than both the Sly treated group and RBC lysis group (Fig. 1C). These observations suggest that hemolytic products, liberated following RBC lysis by Sly, can amplify the damage wrought by Sly on the *in vitro* BBB model. Furthermore, the findings hint that the hemolytic products, originating from RBC lysis instigated by Sly, might harbor compositional disparities compared to those in ultrasonically lysed RBC products, with unidentified components potentially boosting Sly’s proficiency to jeopardize BBB integrity.

### Sly hemolytic products enable SS2 penetration into the BBB model

This investigation sought to elucidate the effect of Sly hemolytic products on the incursion of SS2 into the BBB. Both wild-type SS2 (SS2 WT) and *sly*-deficient SS2 (SS2 Δ*sly*) were co-incubated with RBCs in the upper compartment of the BBB model for a span of 6 h, followed by evaluating the colony-forming units (CFU) of SS2 in the lower chamber. The outcomes revealed a significant amplification of bacterial CFU in the lower chamber in SS2 WT and RBCs groups, compared to the SS2 WT group (Fig. 1D). Conversely, no significant modulation in bacterial CFU in the lower chamber discerned in SS2 Δsly and RBCs group, as opposed to the SS2 Δsly group (Fig. 1D). These insights suggest that Sly predominantly mediates hemolytic activity against RBCs among the virulence factors secreted by SS2. Furthermore, the hemolytic products assume a crucial role during the SS2 invasion into the BBB model.

### Sly hemolytic products exacerbate Sly-induced systemic inflammatory damage

**Fig 2:**
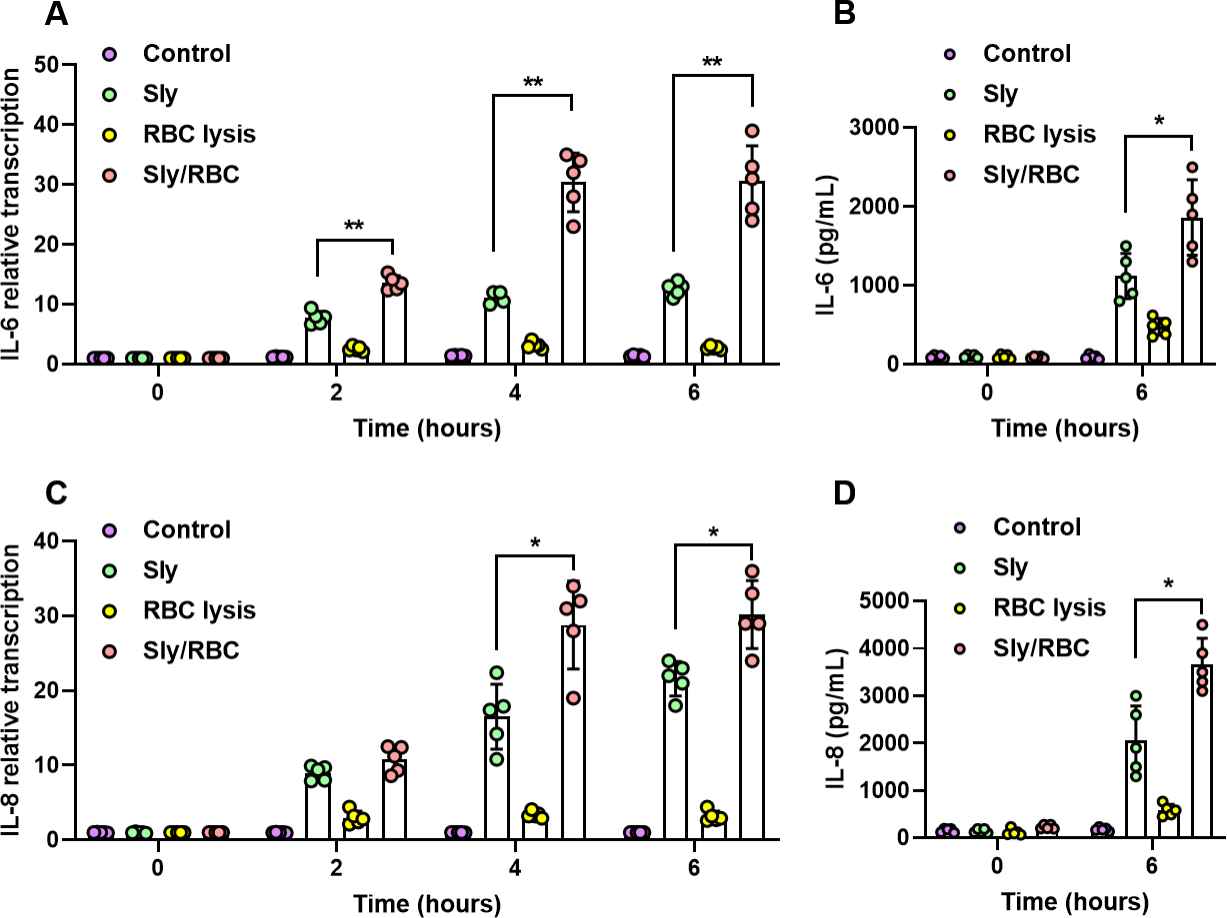
Sly Hemolysin products intensify Sly-induced inflammatory cytokine release from brain microvascular endothelial cells. (**A and B**) Sly hemolysin products escalate Sly-induced IL-6 transcription and expression within hCMEC/D3 cells. (**C and D**) Sly hemolysin products elevate Sly-induced IL-8 transcription and expression within hCMEC/D3 cells. The data are presented as the mean ± standard deviation (SD) and were analyzed by one-way ANOVA. **P < 0.01, *P < 0.05.

Inflammatory responses critically influence BBB impairment during SS2 infection in organisms. To probe this, the present study utilized RT-PCR methodology to scrutinize the impact of Sly and its hemolytic products on the expression of inflammatory cytokines in hCMEC/D3 cells. Observations indicated that, relative to the control group, both the RBC lysis-treated group and the Sly-treated group manifested significantly elevated expression levels of TNF-α (Fig. S1A), IFN-γ (Fig. S1B), IL-6 (Fig. 2A), and IL-8 (Fig. 2C). Moreover, IL-6 and IL-8 in the group treated with Sly and RBCs displayed significantly elevated levels in comparison to the other three groups, implying that Sly hemolytic products can exacerbate Sly-induced elevation in IL-6 and IL-8 expression.

By ELISA, cytokine expression in hCMEC/D3 cells was evaluated at the 6 h juncture across distinct treatment groups. The findings highlighted that, compared to the untreated group, both the RBC lysis-treated group and the Sly-treated group showed significant surges in IL-6 and IL-8 expression. Remarkably, the Sly and RBC treated group demonstrated conspicuously elevated levels compared to the other three groups (Fig. 2B and D). These results are consonant with the RT-PCR data, reinforcing the concept that Sly hemolytic products can amplify the inflammatory damage induced by Sly within the organism.

### IL-33 within Sly hemolytic products augments BBB permeability

**Fig 3:**
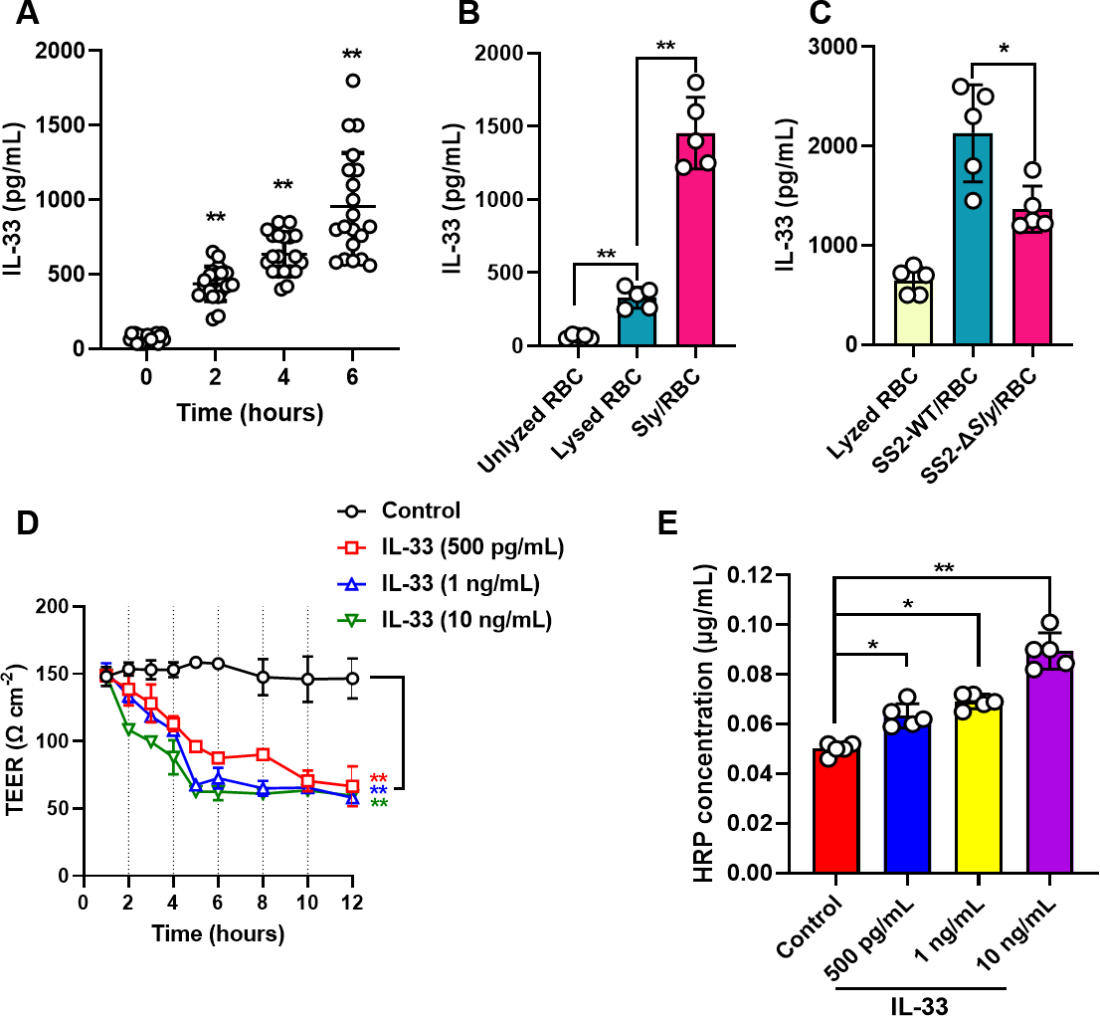
IL-33 originating from Sly hemolysin products can increase the permeability of the BBB model. (**A**) Intravenous injection of Sly increases the concentration of IL-33 in mouse serum. (**B**) Sly lysis of RBC releases a substantial amount of IL-33. (**C**) Knockout of the sly gene reduces the SS2-induced increase in IL-33 release. (**D**) TEER measurement method to assess the impact of IL-33 on the permeability of the BBB model (*n* = 3). (**E**) HRP permeability experiment to evaluate the effect of IL-33 on the permeability of the BBB model. The data are presented as the mean ± standard deviation (SD) and were analyzed by one-way ANOVA. **P < 0.01, *P < 0.05.

Earlier research postulates that Sly hemolytic products harbor elements capable of amplifying BBB permeability. It is acknowledged that products of red blood cell lysis encompass various DAMPs, such as Heme, HSP70, ATP, mtDNA, IL-33, and CypA, each possessing the potential to invoke robust inflammatory responses within the organism (11). Endeavoring to ascertain whether these elements are instrumental in Sly deleterious effects on the BBB, Sly protein was delivered to C57BL/6 mice via tail vein injection, succeeded by evaluations of serum concentrations of the aforementioned DAMPs at diverse post-injection intervals. The data underscored that intravenous Sly injection precipitated an augmentation in serum IL-33 levels in mice, progressively intensifying over time (Fig. 3A).

Assessments of IL-33 expression levels were conducted in the unlyzed RBC group, lyzed RBC group, and Sly and RBC group. Relative to the unlyzed RBC group, the lyzed RBC group revealed an enhancement in IL-33 content, while the Sly and RBC group demonstrated significantly elevated IL-33 content compared to the other two groups. Importantly, the magnitude of IL-33 liberated due to Sly-induced red blood cell lysis was markedly superior to that from ultrasonically lysed RBCs (Fig. 3B). Additionally, when SS2 WT was introduced to RBCs, the subsequent release of IL-33 significantly surpassed that from the SS2 Δsly treatment group (Fig. 3C). In aggregate, these findings imply that Sly facilitates the expression of IL-33 in RBCs. Sly lyses RBCs, releasing a substantial amount of IL-33, and lyse brain microvascular endothelial cells. However, it is unknown whether Sly and its hemolytic products can induce the release of IL-33 from brain microvascular endothelial cells. Therefore, we analyzed the impact of Sly and its hemolytic products on IL-33 in hCMEC/D3 cells. Results indicate that both Sly and its hemolytic products can trigger the release of IL-33 from hCMEC/D3 cells (Fig. S3).

Exploring the impact of IL-33 on the functionality of an *in vitro* BBB model, varying concentrations of IL-33 were administered, with TEER values under continual surveillance. The findings illuminated that IL-33 concentrations of ≥500 pg/mL could appreciably curtail the TEER values of the *in vitro* BBB model (Fig. 3D). CypA also reduces the TEER values in the BBB model (Fig. S2), suggesting that CypA may also participate in the BBB disruption process mediated by Sly hemolytic products. However, compared to IL-33, CypA has a relatively weaker disruptive effect on the BBB model. Therefore, in this study, we primarily focused our research on the interaction between IL-33 and the BBB. Post-IL-33 application to the BBB model for 6 h, a conspicuous amplification in the model permeability to HRP observation. Additionally, this augmentation exhibited a dose-dependent relationship, with HRP permeability escalating in tandem with rising concentrations of IL-33 (Fig. 3E).

### IL-33 compromises tight junction integrity among BMEC

**Fig 4:**
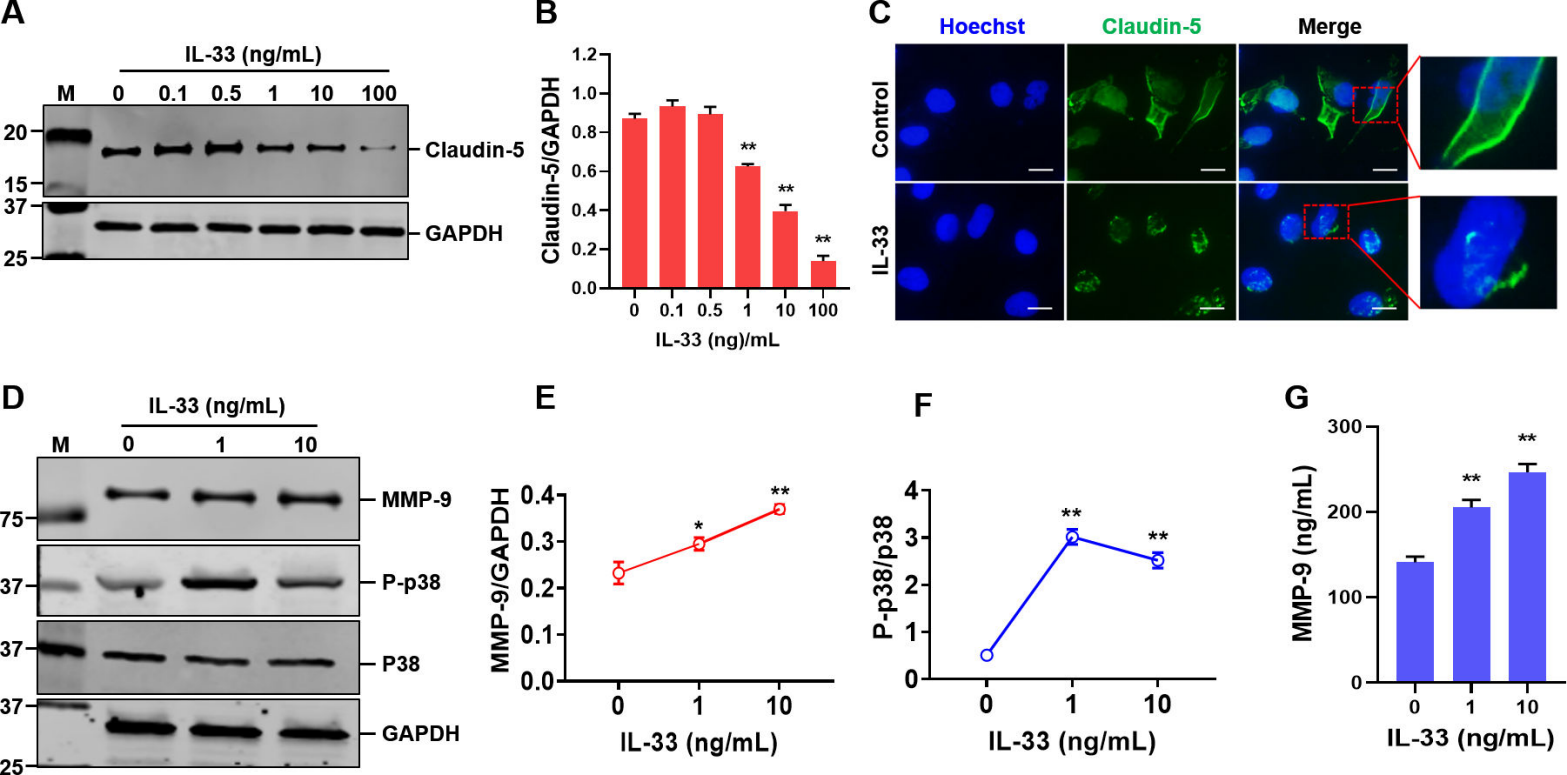
IL-33 Inhibits the expression of tight junction proteins in BMEC and enhances activation of the p38 signaling pathway. (**A**) IL-33 suppresses the expression of Claudin-5 and can cause abnormal distribution of Claudin-5. (**B**) Relative protein expression of Claudin-5. (**C**) Immunofluorescence analysis method is used to examine the expression and distribution of Claudin-5 in hCMEC/D3 due to IL-33. The working concentration of IL-33 is 10 ng/mL. Bar, 10 µM. (**D**) IL-33 promotes the expression of MMP-9 and phosphorylation of p38 in hCMEC/D3. (**E**) Grayscale analysis of the expression of MMP-9. (**F**) Grayscale analysis of the expression of p38, and phosphorylated p38. (**G**) MMP-9 expression in cells treatment with IL-33 was measured using ELISA (*n* = 3). The data are presented as the mean ± standard deviation (SD) and were analyzed by one-way ANOVA. **P < 0.01, *P < 0.05.

The conspicuous diminution of TEER in the IL-33-treated BBB model intimates potential compromise to the tight junction proteins interlinking hCMEC/D3 cells. To validate this proposition, the expression of the intercellular tight junction protein, Claudin-5, scrutinized following the treatment of hCMEC/D3 cells with assorted concentrations of IL-33. Observations revealed that IL-33 concentrations ≥1 ng/mL significantly attenuated Claudin-5 expression, with the inhibitory impact of IL-33 on Claudin-5 expression amplifying concomitantly with its concentration (Fig. 4A and B). Notably, post-IL-33 treatment, hCMEC/D3 cells exhibited not only a decline in Claudin-5 expression levels but also perturbations in its distribution. Whereas Claudin-5 in unaltered cells predominantly coalesces around the cellular periphery, orchestrating tight junctions to establish a barrier, its distribution devolved into an irregular and dispersed pattern within the cells subsequent to IL-33 treatment (Fig. 4C). The results above intimate that IL-33 is capable of inhibiting Claudin-5 expression in cells and perturbing its subcellular localization, thereby potentially undermining the structural integrity of the BBB.

### IL-33 augments MMP-9 expression and catalyzes p38 activation in BMEC

To delve deeper into the molecular mechanisms through which IL-33 escalates permeability in the BBB model, varying concentrations of IL-33 were administered to hCMEC/D3 cells. Subsequently, after 6 h of treatment, the protein expression levels of MMP-9, p-p38 MAPK, and p38 MAPK were gauged (Fig. 4D). The findings illuminated a modest upsurge in MMP-9 expression (Fig. 4E). The expression level of p-p38 in the IL-33 treatment cohorts was elevated compared to the untreated group; however, intriguingly, the expression level of p-p38 protein in the 10 ng/mL IL-33 cohort was less than that in the 1 ng/mL group (Fig. 4F). The overall p38 expression level within the cells remained relatively invariable under the influence of IL-33 (Fig. 4D and F). After treatment with different concentrations of IL-33, MMP-9 expression in cells was measured using ELISA (Fig. 4G).

### IL-33 orchestrates the compromise of the BBB model by Sly hemolytic products

**Fig 5:**
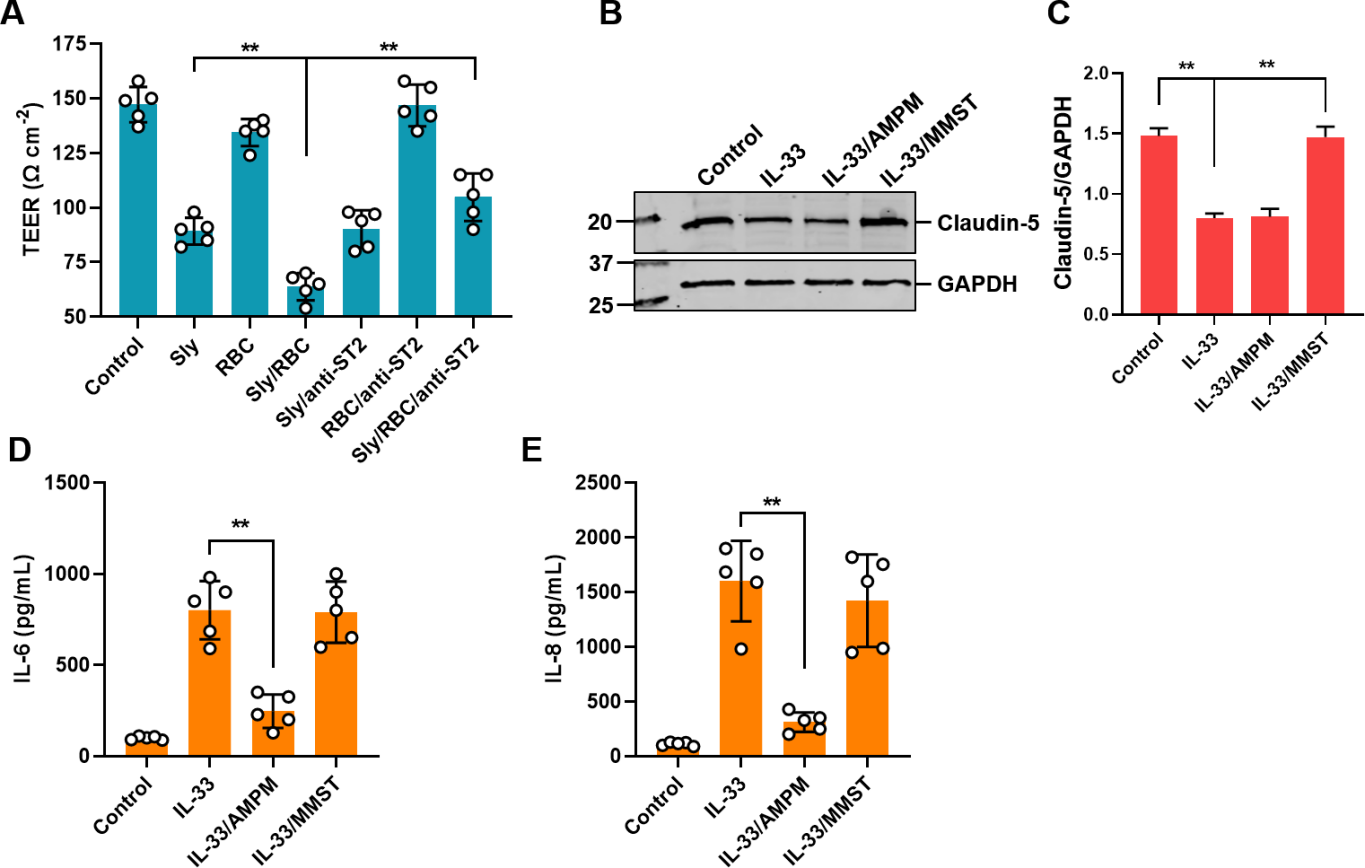
The IL-33/ST2 axis mediates the disruption of the BBB model by Sly hemolytic products. (**A**) TEER method to detect the role of IL-33/ST2 signaling in the process of BBB disruption by Sly hemolytic products. (**B**) MMP-9 mediates the suppression of Claudin-5 expression by IL-33. AMPM, p38 inhibitor Adezmapimod (SB203580). MMST, MMP-9 inhibitor, Marimastat (BB-2516). (**C**) Relative protein expression of Claudin-5. (**D and E**) The p38 signaling pathway mediates the promotion of IL-6 and IL-8 expression by IL-33. The data are presented as the mean ± standard deviation (SD) and were analyzed by one-way ANOVA. **P < 0.01, *P < 0.05.

Earlier investigations unveiled a notable concentration of IL-33 within Sly hemolytic products, wielding the capability to undermine the barrier function of an *in vitro* BBB model. Probing the particular mechanism through which IL-33 mediates the disturbance of the BBB by Sly hemolytic products, the present study subjected the BBB model to Sly and its hemolytic products, juxtaposing the extent of BBB disruption in contexts with and without the incorporation of anti-ST2. Data illustrated that groups treated with Sly, subjected to RBC lysis, and co-treated with both Sly and RBC maintained the capacity to depress the model TEER even following 12 h of exposure to the BBB model. Conversely, the integration of anti-ST2 markedly curtailed the TEER diminution within the model instigated by Sly hemolytic products (Fig. 5A).

### MMP-9 mediates the suppression of Claudin-5 expression induced by IL-33

The investigation plumbs the depths of the prospective involvement of p38 and MMP-9 in the IL-33-elicited inhibition of Claudin-5 expression within hCMEC/D3 cells. The study explores the outcomes of deploying a p38 inhibitor, Adezmapimod (SB203580), and an MMP-9 inhibitor, Marimastat (BB-2516), on the IL-33-driven suppression of Claudin-5. Intriguingly, whereas the p38 inhibitor did not impact IL-33-induced Claudin-5 suppression, the MMP-9 inhibitor mitigated the inhibitory effect of IL-33, amplifying Claudin-5 expression (Fig. 5B and C). This critical insight underscores that MMP-9 acts as a key modulator in IL-33-mediated Claudin-5 suppression within cells, illuminating intricate cellular regulatory mechanisms.

### The p38 MAPK signal pathway mediates IL-33-induced promotion of IL-6 and IL-8 expression in hCMEC/D3 Cells

This section investigates whether p38 and MMP-9 are involved in the IL-33-induced expression of IL-6 and IL-8 in brain microvascular endothelial cells. The study evaluates the impact of adding the p38 inhibitor Adezmapimod (SB203580) and the MMP-9 inhibitor Marimastat (BB-2516) on the IL-33-induced high expression of IL-6 and IL-8 in the cells. The findings reveal that the p38 inhibitor can suppress the IL-33-induced high expression of IL-6 and IL-8, while the MMP-9 inhibitor does not affect their expression (Fig. 5D and E).

### IL-33/ST2 assisted in the destruction of the mouse BBB by SS2

**Fig 6:**
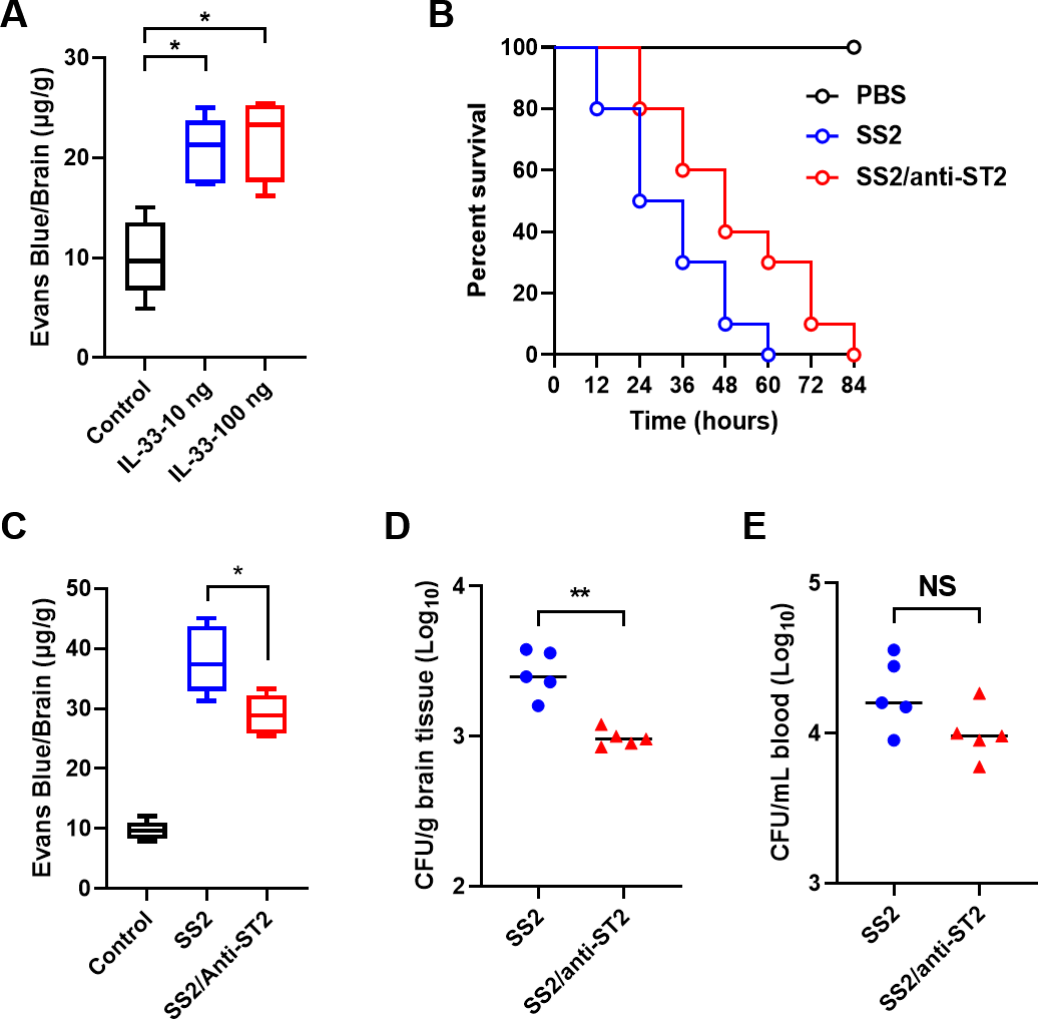
The IL-33/ST2 axis mediates the invasion of SS2 into the mouse BBB. (**A**) Intravenous injection of IL-33 increases the permeability of EB molecules in the mouse brain (*n* = 5). (**B**) Intravenous injection of anti-ST2 delays the death of the acute inflammatory mouse model caused by SS2 infection (*n* = 10). (**C**) Intravenous injection of anti-ST2 reduces the permeability of EB in the brains of SS2-infected mice (*n* = 5). (**D**) Intravenous injection of anti-ST2 reduces the bacterial load in the brains of SS2-infected mice. (**E**) Intravenous injection of anti-ST2 has no effect on the bacterial load in the blood of SS2-infected mice. The data are presented as the mean ± standard deviation (SD) and were analyzed by one-way ANOVA (**A–C**) or Student’s *t* test (**D and E**). **P < 0.01, *P < 0.05.

The *in vitro* findings, demonstrating the potent effect of IL-33 released from Sly hemolytic products on enhancing BBB model permeability, have paved the way for this study aiming to investigate whether IL-33 replicates this effect in an *in vivo* mouse model. Utilizing an intravenous injection method to introduce IL-33 to mice, cerebral permeability to EB meticulously was analyzed. Notably, in stark contrast to the saline-injected control ensemble, IL-33 markedly amplified cerebral permeability to EB in the mice (Fig. 6A), thereby highlighting the influential role of IL-33 in orchestrating BBB integrity.

To probe whether the anti-ST2 antibody alters IL-33 facilitation of SS2 invasion into the brain, mice intravenously injected with anti-ST2, and subsequently faced infection with a lethal dose of SS2. Both survival rate and duration were under scrupulous observation. Fascinatingly, while anti-ST2 failed to amplify the survival rate of the mice, it did significantly prolong the survival duration post-lethal SS2 infection (Fig. 6B).

Simultaneously, BBB permeability to EB scrutinized in SS2-infected mice receiving anti-ST2 injections. Compared to SS2-infected mice administered saline, those receiving anti-ST2 exhibited a considerable contraction in BBB permeability to EB (Fig. 6C). At six hours post-infection, blood and brain tissue samples meticulously were extracted from the mice, under aseptic conditions, for bacterial enumerations. Intriguingly, while SS2-infected mice injected with anti-ST2 revealed a conspicuous reduction in bacterial colonization within the brain (Fig. 6D), the bacterial tallies within the blood remained analogous between both groups (Fig. 6E).

Synthesizing insights from these investigative endeavors, it is palpable that the IL-33 signaling pathway emerges as a critical arbitrator, mediating the deleterious impacts of SS2 on the BBB and facilitating its subsequent encroachment into the brain.

## DISCUSSION

Our results illuminate critical insights into the multifaceted interactions between SS2 and the BBB, underscoring the pivotal role of Sly and its hemolytic products in exacerbating BBB permeability. Notably, we unveiled that Sly, beyond its direct effects, engenders a cascade of inflammatory responses, particularly through the upregulation of IL-33, which subsequently modulates IL-6 and IL-8 expression via the p38 MAPK signaling pathway and impairs Claudin-5 expression through MMP-9, thereby contributing to BBB disruption. The insidious manner through which Sly and its hemolytic products compromise BBB integrity and facilitate SS2 invasion into the brain delineates a nuanced pathogenesis pathway of SS2-induced meningitis.

IL-33, a nuclear cytokine derivative of the IL-1 superfamily, is paramount in maintaining internal homeostasis and immune functionality, wielding a potent influence on type 2 immune responses (25). This regulatory mechanism not only propels parasitic expulsion but also exacerbates inflammatory diseases such as allergies and asthma (15). The elucidation of the type 2 immune response significance in tissue repair and homeostasis has illuminated the critical role of IL-33 in these domains, directing a network of ST2^+^ regulatory T cells, reparative macrophages, and type 2 innate lymphoid cells vital to tissue development and stability (25). Our study demystifies the exacerbation of Sly deleterious impacts on the BBB by Sly hemolytic products, attributing a pivotal role to IL-33 in this paradigm. Mechanistic studies imply IL-33 compromises BBB permeability, undermining the expression of the tight junction protein, Claudin-5, and potentially sparking excessive inflammation in cerebral microvascular endothelial cells. This augments our understanding of IL-33 functionality within this context.

Complex Sly hemolytic products yield varied DAMPs, among which IL-33 identified as a factor heightening BBB permeability. However, anti-ST2 utilization does not substantially ameliorate the TEER reduction, propelling the hypothesis that other components within Sly hemolytic products potentially influence BBB permeability. In our study, we also observed that CypA can diminish the TEER in the BBB model (Fig. S2), laying a foundation for future investigations.

Sly hemolytic products induces augmented expression of IL-6 and IL-8 in brain microvascular endothelial cells. IL-8, a pivotal neutrophil chemotactic factor, facilitating the formation of local neutrophil extracellular traps (NET), instrumental in antibacterial processes (26). Prior investigations highlight that elevated IL-8 alone can amplify BBB model permeability and intravenous IL-8 can elevate brain permeability to EB (27), which may unravel one mechanism through which IL-33 escalates BBB permeability. IL-6, a pro-inflammatory cytokine, can elicit exacerbated inflammatory responses and overstimulate immune cells when expressed in excess. After ischemic stroke, administration of glyceryl triacetate (GTA) resulted in enhanced lipogenesis and exogenous IL-33 upregulation, could lead to improved repair of the BBB damage (28, 29). Our findings suggest that increased IL-33 leads to BBB damage, and we hypothesize that this BBB damage caused by Sly hemolytic products is due to the passive influenced by IL-33, while lipidgenesis-mediated IL-33 upregulation promotes BBB repair is an active regulation by astrocytes in response to BBB damage.

The p38 MAPK signaling pathway enables response to external signals, regulation of inflammation, effectuating varied biological outcomes despite its ostensibly linear architecture. IL-33 markedly circumvents apoptosis via p38 MAPK and AKT pathway activation, a process antagonized by the p38 MAPK inhibitor, SB203580 (30). This research unveils IL-33 promotion of inflammatory cytokines IL-6 and IL-8 release via p38 MAPK pathway activation, a phenomenon suppressed by SB203580, but not ERK inhibitors.

As a critical pathogen precipitating meningitis, SS2 breaches the BBB, instigating brain infections by exploiting virulence factors and compromising host immune defenses. However, the BBB also impedes certain therapeutic drugs, curbing their efficacy. This investigation unveils IL-33 capacity to amplify BBB permeability, facilitating SS2 brain invasion and enabling typically restricted molecules like EB access to the brain. Consequently, IL-33 and its receptor ST2 may surface as crucial targets for SS2 prevention and therapeutic co-administration in meningitis treatments.

In conclusion, the study unveils Sly hemolytic products as exacerbators of Sly-induced BBB damage, spotlighting a crucial role for IL-33, and elucidates the underpinning mechanisms. These revelations not only furnish a critical theoretical scaffold for exploring the BBB breaching mechanisms by SS2 but also broaden our understanding the multifaceted functionalities of IL-33. Our findings mark a significant stride toward unraveling the intricacies of SS2 pathogenicity and pave the way for developing therapeutic interventions that target key molecular players in the progression of SS2-associated neuroinvasive disease. Further research warranted to explore the potential translational application of these findings in devising strategies for mitigating SS2 infection and preserving BBB

## SUPPLEMENTAL MATERIAL

Supplemental material spectrum.00612-24-s0001.docx Fig. S1-S3; Table S1; Original blots for Fig. 4A, 4C, and 5B.

ASM does not own the copyrights to Supplemental Material that may be linked to, or accessed through, an article. The authors have granted ASM a non-exclusive, world-wide license to publish the Supplemental Material files. Please contact the corresponding author directly for reuse.
