# Supplementary material for: Exploring the destructive synergy between IL-33 and Suilysin hemolysis on blood-brain barrier stability
Source: Microbiol Spectr. 2024 Jul 9;12(8):e00612-24. doi: 10.1128/spectrum.00612-24 (PMC11302228; doi:10.1128/spectrum.00612-24)
Supplement: Supplemental material — Fig. S1-S3; Table S1; Original blots for Fig. 4A, 4C, and 5B. [file spectrum.00612-24-s0001.docx]

**Supplementary Figures**


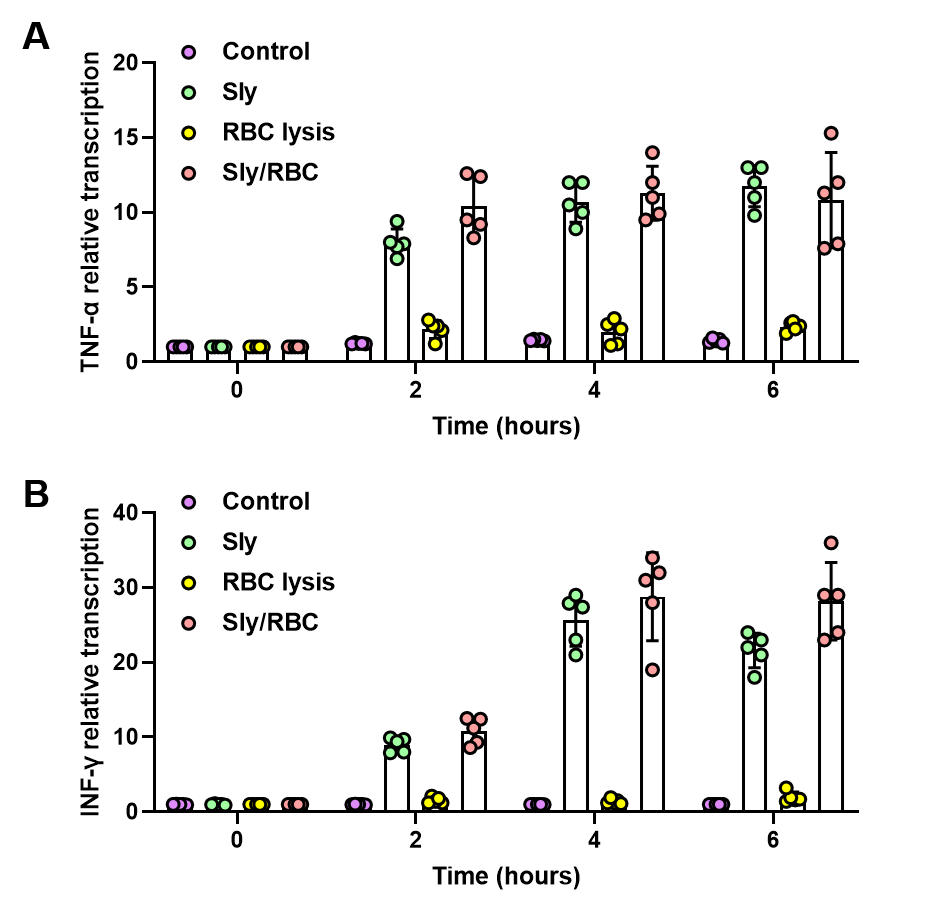


**Fig S1.** **The Effect of Sly Hemolytic Products on TNFα and IFN-γ in Brain Microvascular Endothelial Cells**

Sly promotes the expression of TNF-α(A) and IFN-γ(B) in hCMEC/D3, but the hemolytic products do not affect the expression of both.


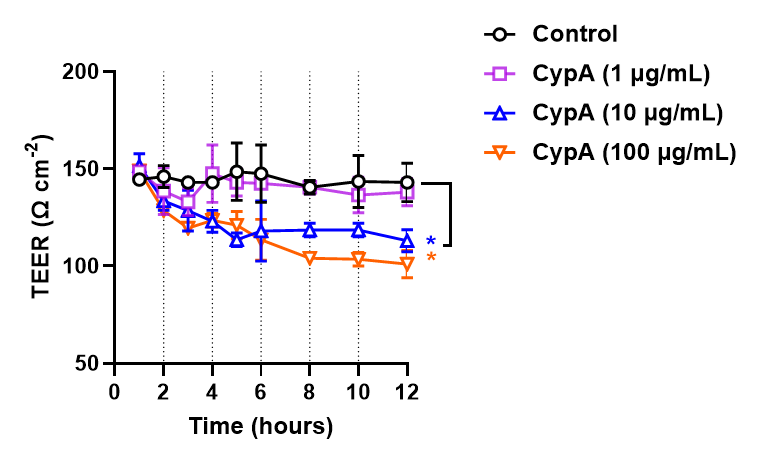


**Fig S2. CypA Can Increase the Permeability of the BBB Model.**

**TEER method to detect changes in BBB permeability.**


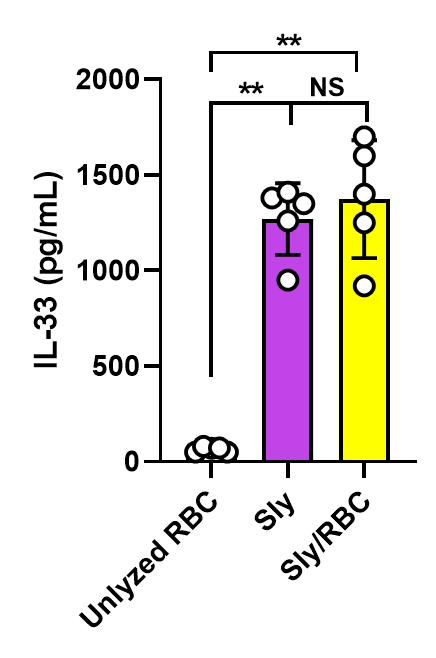


**Fig S3. Sly Can Increase the Expression of IL-33 in Brain Microvascular Endothelial Cells**.

**Orignal Blots**


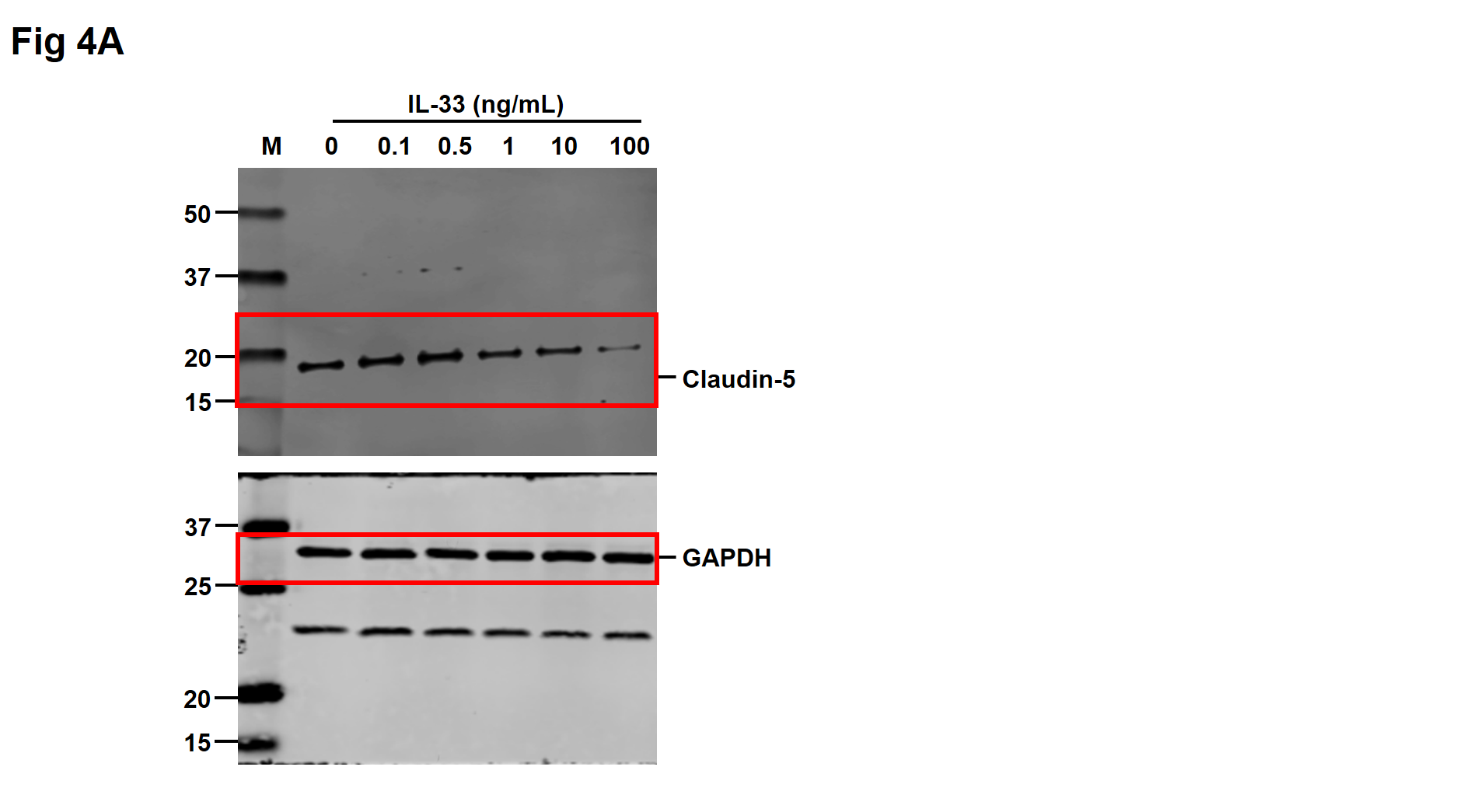


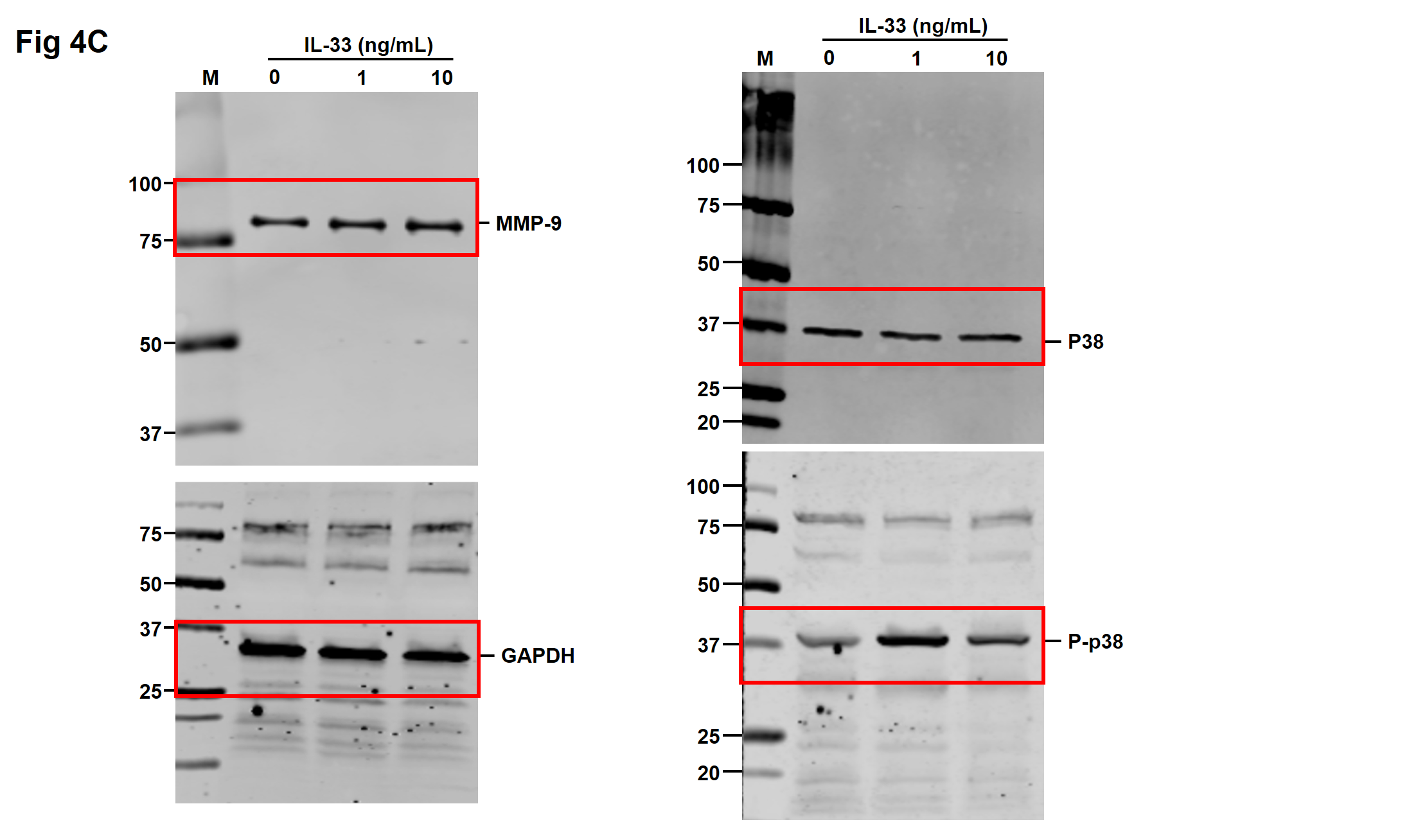


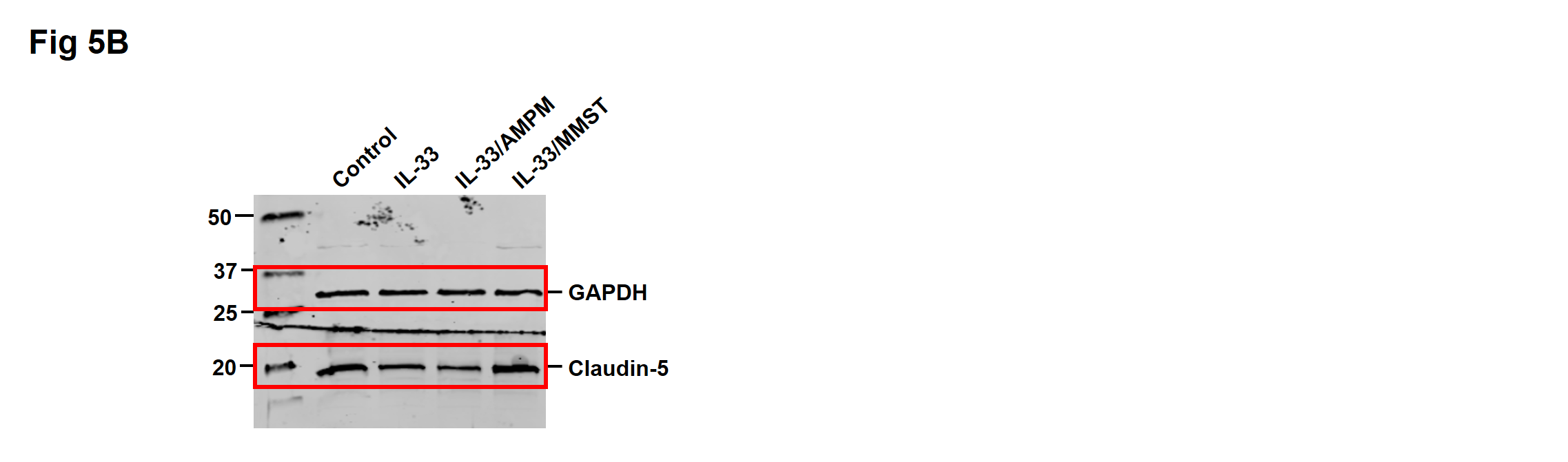


**Supplementary Tables**

**Table S1. The primers for RT-PCR**

| **Gene** | **Sequence** |
| --- | --- |
| *il6* | forward TCAGAACGAATTGACAAACA |
|  | reverse TTGAATCCAGATTGGAAGC |
| *il8* | forward ATGACTTCCAAGCTGGCCGTGGCT |
|  | reverse TCTCAGCCCTCTTCAAAAACTTCTC |
| *ifnγ* | forward TCAGCTCTGCATCGTTTTGG |
|  | reverse GTTCCATTATCCGCTACATCTGAA |
| *tnfα* | forward GGTGATCGGTCCCAACAAGGA |
|  | reverse CACGCTGGCTCAGCCACTC |
| *gapdh* | forward ACCACAGTATGCCATCAC |
|  | reverse TCCACCACCCTGTTGCTGTA |
